# Hematopoietic cell activation in the subventricular zone after Theiler's virus infection

**DOI:** 10.1186/1742-2094-5-44

**Published:** 2008-10-15

**Authors:** Gwendolyn E Goings, Adriana Greisman, Rachel E James, Leanne KF Abram, Wendy Smith Begolka, Stephen D Miller, Francis G Szele

**Affiliations:** 1 Department of Physiology, Anatomy, and Genetics, Oxford University, UK; 2 Department of Pediatrics, Feinberg School of Medicine, Northwestern University, Chicago, IL, USA; 3 Program in Neurobiology, Children's Memorial Research Center, Children's Memorial Hospital, Chicago, IL, USA; 4 Department of Microbiology and Immunology, Feinberg School of Medicine, Northwestern University, Chicago, IL, USA; 5 Interdepartmental Immunobiology Center, Feinberg School of Medicine, Northwestern University, Chicago, IL, USA

## Abstract

**Background:**

The periventricular subventricular zone (SVZ) contains stem cells and is an area of active neurogenesis and migration. Since inflammation can reduce neurogenesis, we tested whether Theiler's murine encephalomyelitis virus (TMEV) induces inflammation and reduces neurogenesis in the SVZ.

**Methods:**

We performed immmunohistochemistry for the hematopoietic cell marker CD45 throughout the central nervous system and then examined neuroblasts in the SVZ.

**Results:**

CD45+ activation (inflammation) occurred early in the forebrain and preceded cerebellar and spinal cord inflammation. Inflammation in the brain was regionally stochastic except for the SVZ and surrounding periventricular regions where it was remarkably pronounced and consistent. In preclinical mice, SVZ neuroblasts emigrated into inflamed periventricular regions. The number of proliferating phoshpohistone3+ cells and Doublecortin+ (Dcx) SVZ neuroblasts was overall unaffected during the periods of greatest inflammation. However the number of Dcx+ and polysialylated neural cell adhesion molecule (PSA-NCAM+) SVZ neuroblasts decreased only after periventricular inflammation abated.

**Conclusion:**

Our results suggest that after TMEV infection, the SVZ may mount an attempt at neuronal repair via emigration, a process dampened by decreases in neuroblast numbers.

## Background

The two regions of the brain most intensively scrutinized in recent years for their reparative or cell replacement potential are the subventricular zone (SVZ) which lines the lateral ventricles and the subgranular zone (SGZ) of the hippocampal dentate gyrus [1,2]. Both regions daily generate thousands of interneurons that integrate into synaptic circuitry. SVZ neuroblasts migrate long distances from their birthplaces near the lateral ventricles to the olfactory bulbs via the rostral migratory stream (RMS) [3]. The SVZ, including human SVZ, contains cells that self-renew and are multipotential when exposed to appropriate growth factors in vitro; they are stem cells [4]. As such, they may provide a source for cell replacement, and many experiments have shown they attempt repair of damaged or diseased tissue [1].

Hipppocampal microglia dampen neurogenesis during inflammation [5,6]. Hematopoietic lineage cells comprise approximately five percent of cells in the SVZ [7-9], yet the constitutive and pathological role of these cells within the SVZ is poorly understood. SVZ microglia are distinctive: they express relatively high levels of CD45, a tyrosine phosphatase, they proliferate more than microglia in non-neurogenic regions, and are resistant to traumatic brain injury that causes microglial activation in adjacent nuclei [7]. Macrophages migrate into the brain during late development in certain foci, including the lateral ventricles and SVZ [10] and then become resident microglia. Interestingly, neural macroglia (astrocytes and oligodendrocytes) are generated in the SVZ during late development and migrate throughout the forebrain. Thus although microglial and macroglial lineages are different [11], their emigration routes from the SVZ into the postnatal forebrain are very similar. These same migratory routes, fanning out into the forebrain from the SVZ, are followed by SVZ neuronal cells after a variety of injuries and diseases [12,13].

CD45 exhibits multiple splicing isoforms and modulates microglial and T cell activation [14-16]. Upon activation, resident microglia undergo morphological changes consistent with their function: amoeboid for migrating to areas of injury and round for phagocytosis. FACsorting of immunofluorescent cells is used to distinguish resident microglia (CD45^low^) from infiltrating macrophages (CD45^high^) [17]. We have shown that immunolabelling sections with anti-CD45 antibodies also reveals CD45^low ^(the majority of cells under normal conditions) versus CD45^high ^cells [7]. Levels of CD45 expression correlate with levels of microglial activation, CD45^high ^expression indicating significant activation. Interestingly, CD45 mutations have been observed in some multiple sclerosis (MS) patients suggesting that it is not merely a "marker" but may contribute to the etiology of the disease [18].

MS is a demyelinating disease mediated by inflammation. In addition to loss of oligodendrocytes and myelin, neuronal apoptosis occurs in the forebrain and other regions [19-21]. The etiology of the disease is still elusive; possibilities range from spontaneous autoimmunity to a primary CNS insult such as infection. Because of the variability and uncertain etiology of MS, a variety of preclinical models are used to replicate specific features of the disease. The sclerotic lesions evident in the CNS are sites of CD45+ microglial, macrophage, dendritic cell, and T-cell accumulation indicative of an inflammatory response. Theiler's murine encephalomyelitis virus (TMEV) infection of susceptible SJL mice is a model used to study this response [22-24]. The TMEV model is consistent with chronic, progressive inflammatory demyelination rather than the relapsing/remitting disease profile of the experimental autoimmune encephalomyelitis (EAE) model [25]. In addition, TMEV results in the direct infection of microglia, whereas in EAE, microglia are activated secondary to the autoimmune response [25].

Interestingly the two co-receptors used by different Theiler's virus strains, sialic acid and heparan sulfate [26] are expressed at high levels in the SVZ. In fact, polysialic acid residues attached to the neural cell adhesion molecule (PSA-NCAM) are required for SVZ neuroblast migration [27]. The SVZ augments new oligodendrocyte production in EAE [28,29], and syngeneic SVZ neurospheres ameliorated EAE pathology in mice, pointing to the potential for SVZ repair of MS [30]. The clinical MRI and pathological description of "Dawson's fingers", lesions extending from the lateral ventricles to surrounding regions [31,32] suggest that the SVZ and periventricular regions are particularly sensitive to MS. Despite these tantalizing data, whether TMEV induces inflammation in the SVZ, and the effects of TMEV on SVZ neurogenesis and migration have remained unstudied until now. If SVZ inflammation is prominent in MS and this reduces neurogenesis, it may reduce autologous repair. Also, though there is an extensive literature on TMEV, a within-study comprehensive characterization of the time-course and anterior to posterior spread of viral induced CD45+ cell activation has not been carried out. Therefore, in these experiments we examined CD45+ cell activation and studied its spatio-temporal relationship to SVZ neurogenesis and emigration after TMEV. We show here with an antibody that recognizes all isoforms of CD45, that forebrain CD45+ cell activation precedes spinal cord activation and that the SVZ is the area with the most consistent CD45+ cell activation. We also document SVZ neuroblast emigration into inflamed periventricular regions and a delayed decrease in neurogenesis.

## Methods

### Mice

Eighty 6–7 week old female wild type SJL/J mice (Taconic Labs) were used in the TMEV studies. All mice were housed in the Northwestern University animal care containment facility and were provided with unlimited access to standard laboratory food and water. Easier access to food and water was provided for TMEV injected animals exhibiting neurological impairment.

### TMEV disease induction

Three groups of mice were used: TMEV, sham, and naïve. Together, sham and naïve mice constituted "controls". TMEV and sham mice were anesthetized with 4% isoflurane. 3 × 10^6 ^PFU BeAN 8386 virus, suspended in sterile 0.03 ml BSS, was injected into the right cerebellar cortex through a 27 gauge needle fitted with a needle guard to prevent penetration beyond 3.5 mm ventral to skull. Injections were localized to a point half-way to midline at ear level. Sham mice received injections of 0.03 ml BSS. Naïve mice were not anesthetized or injected. All mice were monitored for changes in neurological status two to three times per week. Mice were assigned numerical scores as follows: 0 = asymptomatic; 1 = mild waddling gait; 2 = moderate waddling gait without spastic paralysis; 3 = severe waddling gait with mild spastic paralysis; 4 = severe waddling gait with moderate to severe spastic paralysis; 5 = total hind limb paralysis; 6 = moribund. Mice were further divided into three groups according to clinical scores or time point match for sham and naive: preclinical = prior to onset of any symptoms but after initial inflammatory/increased stress due to injections (D 14–24); early onset = clinical score of 1 or more for 2 consecutive days (D 42–47); chronic = increased clinical score (2 or 3) for minimum of 5 consecutive days (D 90). Each group had its respective control mice. Preclinical sham N = 4, Preclinical naïve N = 1, Preclinical TMEV N = 27; early onset sham N = 5, early onset naïve N = 1, early onset TMEV N = 25; chronic sham N = 4, chronic naïve N = 1, chronic TMEV N = 4.

#### Cuprizone induced demyelination

Chow containing 0.2% cuprizone (Harlan Teklad) was fed to C57Bl mice (N = 7) *ad libitum *for 3 weeks. Control mice (N = 7) received the same chow minus cuprizone.

### Tissue preparation and immunohistochemistry

Mice were perfused with 4% paraformaldehyde, brains post-fixed overnight, and cryoprotected in 30% sucrose overnight at 4°C before sectioning. Free-floating coronal sections, 30 μm thick, were cut on a sliding microtome and stored in cryoprotectant at -20°C. Antibodies used: rat anti-CD45 (clone IBL-5/25; 1:500, Chemicon, Temeluca, CA); goat anti-doublecortin (C-terminus; 1:200, Santa Cruz Biotechnology, Santa Cruz California), rabbit anti-BeAn (1:600, Miller Lab, Northwestern University), mouse anti-PSA-NCAM (1:500, Chemicon), rabbit anti-phosphohistone3 (1:500, Upstate Biotechnology). Sections were washed and blocked with 50 mM glycine in phosphate buffered saline (PBS) to reduce autofluorescence of paraformaldehyde-fixed tissue. Sections were washed, blocked in PBS containing 0.1% Triton X-100 and 10% Donkey Serum, DS (Sigma), (PBS+), incubated overnight at 4°C in primary antibodies, washed, incubated one hr at RT in Cy2 (1:200) or Cy3 (1:500) conjugated anti-primary secondaries, and rinsed in phosphate buffer (PB). Sections were mounted and slides were coverslipped with FluorSave mounting medium (Chemicon) in PBS. Ommission of primary antibody was used as controls in all immunohistochemistry experiments.

### Microscopy, quantification, and data analysis

A comprehensive anterior to posterior set of brain sections were examined at the following anatomical coordinates [33]. Olfactory bulb (4.0 to 3.0 mm anterior to bregma), anterior cortex (3.0 to 2.0 mm anterior to bregma), striatum (1.7 to -0.5 mm from Bregma), hippocampus (-1.0 to -2.5 mm posterior to bregma), cerebellum (-5.6 to -7.0 mm from bregma). In addition, we collected sections from cervical, thoracic, and lumbar spinal cord. Immunohistochemistry was examined and recorded on a Leica DMIRB microscope using Openlab software and on a Zeiss Meta confocal microscope, and analyzed in 3-D with Zeiss and Volocity Software. Images were composed in Adobe Photoshop. *Doublecortin SVZ neurogenesis quantification. Doublecortin Cell Counts*. Images of doublecortin and DAPI labeled sections of the dl SVZ were taken at 63× on a Zeiss Meta confocal microscope with a single scan of both channels. The images were saved and exported as tiff images into Openlab Image software (Improvision). In Openlab Dcx+ cells and DAPI+ cell nuclei in the dl SVZ were counted. *Dcx immunofluorescence surface area measurements*. We used these measurements to confirm Dcx+ cell counts. Images of doublecortin labeled sections were taken at identical camera settings and positive Dcx immunofluorescence intensity threshold levels pre-determined. We used these levels to calculate the percent of the SVZ surface area occupied by positive Dcx immunofluorescence in the dorsolateral SVZ (dl SVZ). *Hippocampal Dcx+ cell counts*. All Dcx+ cells were counted in the subgranular zone of the dentate gyrus unilaterally. *Emigrated Dcx+ cell quantification*. The large majority of cells that emigrated in controls and after TMEV did not move more then a few hundred microns from the SVZ. Therefore the large majority of cells were within the distances sampled. Doublecortin labeled striatal sections were viewed at 40× on a Leica DMIRB upright microscope. Emigrated Dcx+ cells were counted unilaterally. With the SVZ to the left margin of the field of view at 40×, Dcx+ cells in the striatum were counted, with the SVZ to the right margin of the field of view, septal Dcx+ cells were counted, with the SVZ in the middle of the field showing the full extent of the CC thickness, Dcx+ cells in the CC were counted, with the ventral 3rd of the SVZ in the middle of the field, emigrated Dcx+ cells in the ventral 3rd septum, striatum and surrounding ventral SVZ were counted. *Phosphohistone3 cell counts*. Images of phosphohistone3 labelled sections of the dorsolateral, medial septal and ventral-medial SVZ and were taken at 40× on a Leica DMRIB microscope. The images were saved and exported as tiff images into Volocity (Improvision). The number of phosphohistone H3+ cells were counted and averaged for 6 consecutive sections per animal ranging from ~bregma 0.9 to 0.0. Only cells that had bright, complete labelling in the nuclei were included, cells that showed fragmented staining were excluded. All measurements were taken by staggering controls and treated mice to avoid quantification drift bias. Satistics were performed in Microsoft Excel; Student's T-tests (two tailed, equal variance) with p values < 0.05 were considered to be significant.

## Results

### TMEV induces CD45+ cell activation in the forebrain before the spinal cord

**Figure 1:**
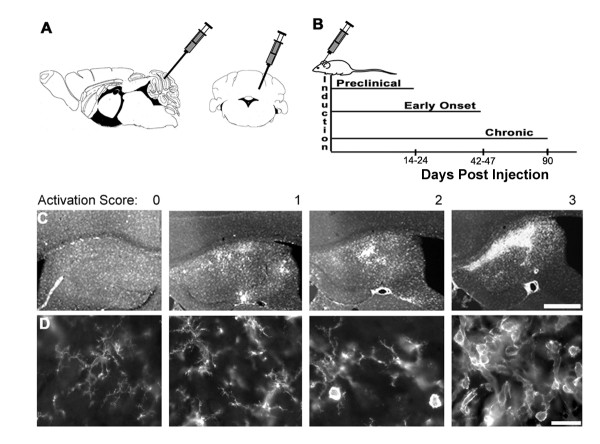
**TMEV injections, timing of experimental groups, and scoring of CD45+ activation.** A) Schematic showing injection site in cerebellum, both sagittal and coronal views shown. Adapted from the atlas of Paxinos and Franklin [33]. B) Time line of experimental regimen. C) Examples of representative CD45+ cell activation showing scores of 0 through 3 in the hippocampus. Scale bar = 100 μm. D) Cells in spinal cord grey matter showing range of morphology of CD45+ cells from non-activated (0) to highly activated (3) with intermediate morphology in between. Scale bar = 10 μm.

TMEV was injected in mice into the left cerebellar cortex (Fig. 1A). After infections, mice were sacrificed at 14–24 days (preclinical, PC), 42–47 days (early onset, EO), and 90 days (Chronic, C) (Fig. 1B), based on behavioural criteria as described in the Methods section. Inflammation and hematopoietic cell activation was scored semi-quantitatively using a 4 point scale based upon cell morphology, cell density, and brightness of CD45+ immunofluorescence (Fig. 1C,D). This was done primarily to allow a within-study comparison of hematopoietic cell activation across regions and time. 0: weakly labeled CD45+ cells (CD45^low^) with highly ramified processes. CD45+ cells are evenly distributed throughout parenchyma. 1: CD45^low ^microglia became CD45^medium ^or CD45^high ^and changed from highly ramified to cells with larger soma and shorter, thicker processes. A score of 1 was characterized by relatively few round CD45^high ^cells that were dispersed randomly. 2: intermittent focal areas of high density CD45^high^, round cells. 3: multiple focal and/or contiguous areas of high density CD45^high^, round cells, with or without large, irregularly shaped cells. Each stage showed a corresponding decrease of the previous stage's morphology and relative CD45+ intensity (Fig. 1C,D).

#### CD45 activation in forebrain

**Figure 2:**
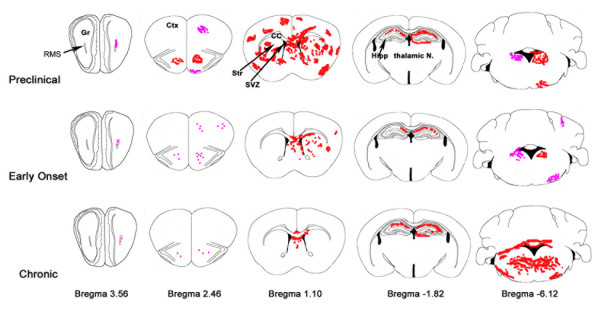
**Schematic of brain regions showing typical areas of CD45+ cell activation. **Shown are qualitative averages of intensity and location of CD45+ cell activation. Pink represents levels 0–1 (low activation), and red, levels 2–3 (high activation). Note the preclinical group shows greater activation compared to early onset and chronic groups, except in the cerebellum where activation is greatest in the chronic group. Anterior – posterior distances are given as distances from bregma. Gr = granular layer of olfactory bulb, RMS = rostral migratory stream, Ctx = cerebral cortex, Str = striatum, SVZ = subventricular zone, CC = corpus callosum, Hipp = hippocampus. Modified from Paxinos and Franklin [33].

Control mice contained an even distribution of CD45^low ^cells throughout the central nervous system (left-most panels in Fig. 1C,D). Sham animals occasionally showed unilateral "hot spots" of CD45 activation in the forebrain which were associated with few amoeboid or round CD45+ cells. This was rare and did not reach a score of 1; naïve animals showed minimal CD45 activation (Table 1). Most CD45+ cells in control mice had many branching processes and exhibited CD45^low ^expression [7]. The forebrain of preclinical TMEV mice showed cell activation already 14 days after TMEV injections and this continued through D24 (Fig. 2, Table 1). Bright CD45+ cell activation often surrounded blood vessels (Fig. 4A). Many CD45+ cells were large, and irregularly shaped; they appeared to be amoeboid. The preclinical group had the greatest amount of activation in the forebrain compared to early onset and chronic groups (Fig. 2, Table 1). This was true throughout most of the forebrain; in the cerebral cortex, striatum, amygdala, and thalamus (Fig. 2, Table 1). The hippocampus, was an exception, showing the greatest CD45+ cell activation in chronic sections (Table 1).

**Table 1:** CD45+ cell activation.

	**Shams**	**Preclinical**	**Early Onset**	**Chronic**
Olfactory Bulb	0.7 ± 0.1	1.6 ± 0.5	1.0 ± 0	1.5 ± 0.3

Anterior cortex	0.4 ± 0.1	2.2 ± 0.6	1.7 ± 0.3	1.3 ± 0.3

Meninges	0.9 ± 0.1	2.2 ± 0.6	1.7 ± 0.3	1.5 ± 0.3

SVZ	0.2 ± 0.1	2.3 ± 0.5	0.7 ± 0.3	0.5 ± 0.5

Striatum	0.9 ± 0.1	2.4 ± 0.4	1.3 ± 0.3	1.0 ± 0

Septum	0.2 ± 0.1	2.3 ± 0.5	0.7 ± 0.3	0.3 ± 0.3

Hippocampus	0.1 ± 0.1	1.8 ± 0.6	2.0 ± 1.0	2.6 ± 0.4

Choroid Plexus	0.8 ± 0.2	2.8 ± 0.2	2.0 ± 0.6	0.9 ± 0.1

3rd Ventricle	0.6 ± 0.2	2.6 ± 0.2	2.0 ± 1.0	2.0 ± 0.4

Fimbria/dorsal fornix	0.6 ± 0.2	2.2 ± 0.6	1.3 ± 0.9	2.0 ± 0.7

Amygdala	0	1.2 ± 0.7	0.3 ± 0.3	0

Thalamus	0	1.2 ± 0.7	0.3 ± 0.3	0.8 ± 0.3

Cerebellum	0	1.4 ± 0.4	1.3 ± 0.7	3.0 ± 0

Cervical Spinal Cord	0	2.0 ± 0.4	2.0 ± 1.0	3.0 ± 0

Thoracic Spinal Cord	0	2.2 ± 0.5	2.0 ± 1.0	2.5 ± 0.3

Lumbar Spinal Cord	0	2.4 ± 0.6	1.7 ± 0.3	2.5 ± 0.3

CD45 activation semi-quantitative scoring summary from olfactory bulb through lumbar spinal cord using a 0–3 scoring system with a score of 3 being the greatest activation. See Fig. 1 for representative images.

#### CD45 activation in cerebellum and spinal cord

The preclinical to chronic gradation was reversed in the cerebellum and brainstem, with chronic mice showing the greatest activation (Fig. 2, Table 1). The primary sites of high activation were the deep cerebellar, vestibular, and reticular nuclei. In addition, CD45+ cell activation was apparent adjacent to the fourth ventricle and in the cerebellar commissure. Surprisingly, very little activation was seen in the cerebellar cortex, the site of viral injection.

**Figure 3:**
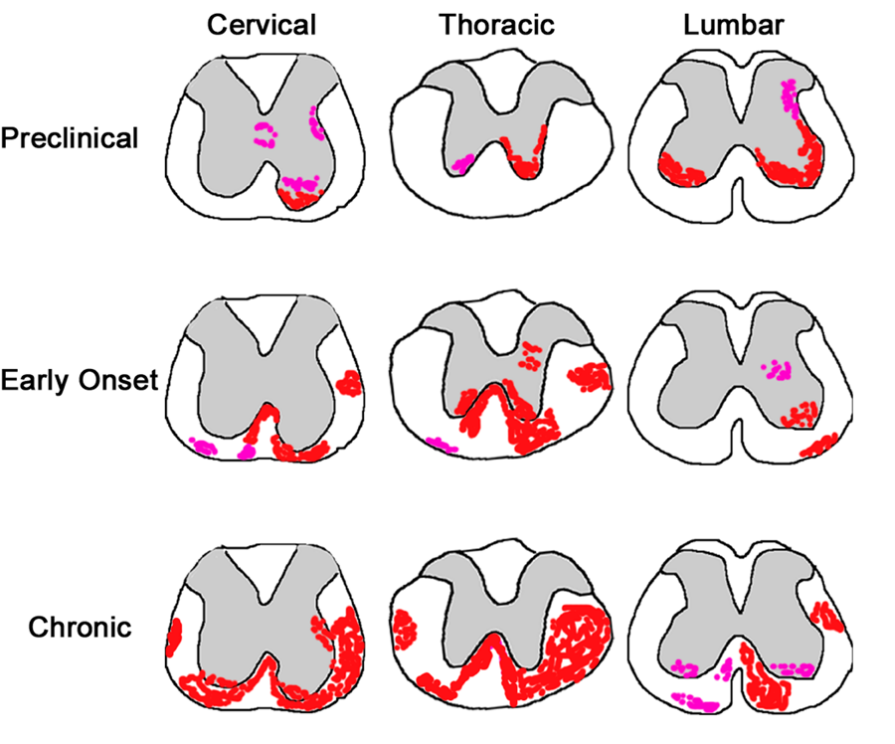
**Schematic showing areas of typical CD45+ cell activation in the spinal cord.** Varying levels of activation are shown in preclinical, early onset, and chronic groups. Pink represents levels 0–1 and red, levels 2–3. Grey matter is indicated by grey shading. Spinal cord images are traces of actual early onset cervical, thoracic and lumbar spinal cord images.

**Figure 4:**
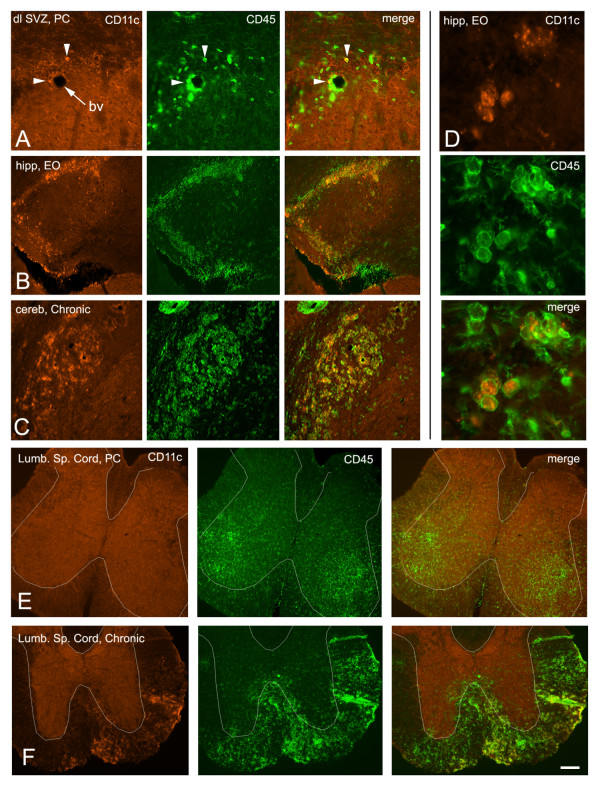
**Comparison of dendritic cell CD11c immunolabelling with CD45 expression.** A) Relatively few CD45+ cells are CD11c+ (arrowheads) in the SVZ. Bv = blood vessel. B, C) A much larger proportion of CD45+ cells are CD11c+ in the hippocampus and cerebellum. D) Cytoplasmic versus membrane labelling of CD11c and CD45, respectively. E) No CD11c+ cells in the lumbar spinal cord of a preclinical mouse, even in areas of CD45 activation. White outlines of the approximate border between grey and white matter were facilitated by the non-specific background staining in the gray matter (orange). F) CD11c+ dendritic cells were a subset of CD45+ cells in lumbar spinal cord white matter of chronic mice.

Similar to the cerebellum, the spinal cord generally followed a preclinical to chronic increase in CD45+ cell activation (Fig. 3, Table 1). The greatest activation in cervical and thoracic sections was at the latest time point. Lumbar spinal cord showed similar levels of CD45+ cell activation at all time points. The spinal cord CD45+ cell activation was also characterized by a distinct shift from grey matter to white matter (Fig. 3, Fig. 4E,F). This pattern of activation occurred at all rostrocaudal positions. In preclinical mice, only grey matter contained activated CD45+ cells. At the early onset time points CD45+ cells were found in both grey and white matter. CD45+ cell activation was predominantly located in the white matter in chronic mice. The majority of activation was in the ventral half of the spinal cord. The cervical spinal cord was the only region to show any activation immediately surrounding the ventricle (Fig. 3).

Myeloid dendritic cells have been implicated in antigen presentation in the EAE model of MS [34]. In addition the CD11c-GFP transgene is expressed at relatively high levels in neurogenic zones of the intact brain [35]. Therefore we examined the expression of the dendritic cell marker CD11c and compared it to CD45. CD11c+ dendritic cells were seen only in areas of CD45^high ^activation in both brain and spinal cord (Fig. 4). Gross examination of CD11c+ cells as a subset of CD45+ cells indicated varying ratios ranging from approximately 10% to 90% of CD45+ cells. Few CD11c+ cells were evident in the pre-clinical spinal cord with CD45 activation in gray matter (Fig. 4E); however many CD11c+ cells were seen in the white matter of the chronic spinal cord, paralleling CD45^high ^activation (Fig. 4F).

### TMEV causes CD45+ cell activation in the SVZ

**Figure 5:**
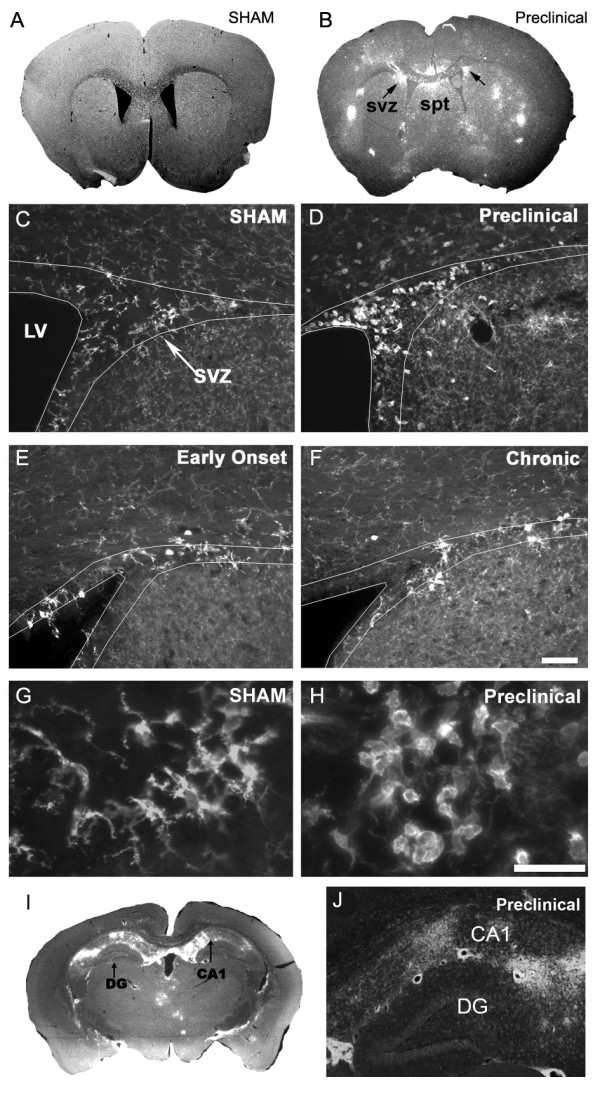
**CD45 immunohistochemistry shows hematopoietic cell activation in the subventricular zone.** A) CD45 expression is barely detectable at low magnifications in control mice. B) Example of CD45 immunohistochemistry in a preclinical mouse. CD45+ cell activation was sporadic throughout the forebrain, but was consistent in the SVZ and septum. svz = subventricular zone, spt = septum. C) Sham mouse showing ramified microglia typical in all control animals. D) Preclinical mouse showing round, activated CD45^high ^microglia. E, F) are early onset and chronic experimental sections. Early onset section shows a mixed population of ramified, intermediate morphology and round microglia. Ramified and intermediate morphologies of microglia predominate in the chronic SVZ. Scale bar C-F = 50 μm. SVZ in C-F outlined based on high density of cells in the SVZ detected with DAPI nuclear staining. G) Higher magnification of ramified microglia in a sham animal. H) Higher magnification from an experimental animal. Scale bar G, H = 20 mm. I, J) CD45 cell activation in the hippocampus is restricted to the CA regions and excluded from the dentate gyrus.

**Figure 6:**
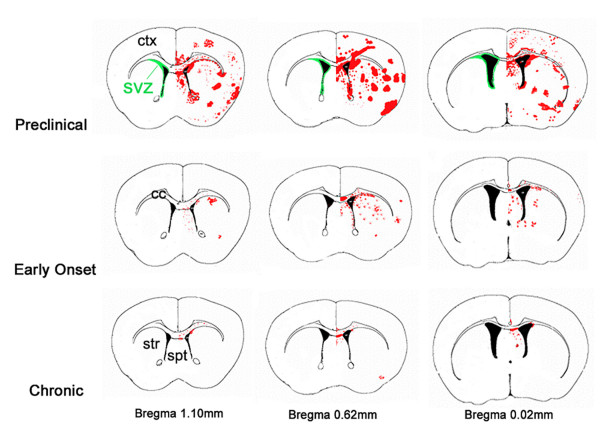
**Schematic of typical CD45 activation at three anterior to posterior sections through the SVZ in preclinical, early onset and chronic groups.** A unilateral view is shown although the activation was bilateral. The SVZ is indicated in green. Anterior posterior distances from bregma are indicated.

In examining CD45+ cell activation, we noted that the specific location varied from mouse to mouse, and from section to section. In contrast, CD45+ activation was very consistent in the SVZ and immediately surrounding regions (Figs. 5, 6). Unlike other brain regions that normally exhibit CD45^low ^levels, SVZ cells seem to be constitutively "semi-activated": they have ramified processes and express CD45 at medium (and occasionaly high) levels [7] (Fig. 5C,G). At preclinical TMEV stages, most CD45+ SVZ cells became activated; they were round and CD45^high ^(Fig. 5B,D,H). CD45^high ^cells in and around the SVZ were frequently associated with blood vessels that had perivascular cuffs of activation. SVZ CD45^high ^cells were seen the length of the lateral ventricles, and were prominent in the ventral SVZ (Fig. 2, Fig. 5B, Fig. 6) an area that exhibits a high degree of neurogenesis [36]. Though CD45+ cell activation in the SVZ was present at all time points, the preclinical group had greatest activation and the chronic group the least (Fig. 5D–F, Fig. 6). Interestingly, CD45+ cell activation was also consistently high in the rostral migratory stream as it courses through the anterior cerebral cortex into the olfactory bulb (Fig. 2). Similar to the SVZ, this was characterized by round CD45^high ^cells and was most prominent in preclinical mice.

CD45 cell activation also consistently occurred in regions close to the SVZ; the dorsal septum and striatum. Similar to the rest of the forebrain, this occurred most prominently in preclinical mice, but was also apparent in early onset and chronic mice (Fig. 2, Fig. 5B, Fig. 6). The corpus callosum, immediately dorsal to the SVZ, also contained activated CD45+ cells however, to a lesser extent than grey matter areas (Fig. 5B). We next examined whether consistent CD45+ cell activation occurred in the other neurogenic region of the forebrain, the dentate gyrus of the hippocampus. The majority of activity was in the dorsal hippocampus subjacent to the lateral ventricle, sandwiched between it and the corpus callosum (Fig. 2, 5I,J). CD45+ cell activation was evident in the CA1, CA2, pyramidal and oriens layers of the hippocampus, however it was notably absent from the dentate gyrus (Fig. 2, 5I,J). Nearby, CD45^high ^cell activation was observed in the dorsal fimbria and consistently in the 3^rd ^ventricle (Fig. 2, Table 1).

**Figure 7:**
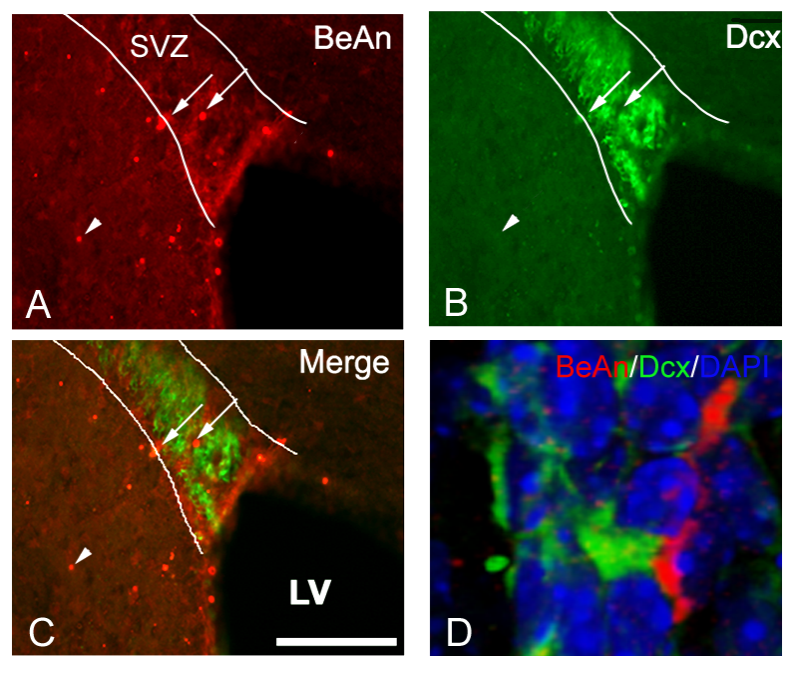
**TMEV colabels with Dcx+ and CD45+ cells in the SVZ and periventricular regions. **A) Dorsal SVZ labelled with anti-BeAn immunohistochemistry in preclinical sections. Note viral immunolabelling (arrows) in SVZ (outlined based on DAPI staining) and in surrounding parenchyma (arrowheads). Some viral particles were seen in Dcx+ cells. B) Dcx immunolabelling in same section. C) Merge of BeAn and Dcx immunolabelling. D) 3-dimensional view of BeAn immunolabeling. Arrow shows BeAn immunopositive profile closely associated with a Dcx+ cell.

TMEV infects macrophages but has recently been proposed to target polysialic acids [26,37]. The SVZ is the forebrain region with the highest concentration of polysialylated neural cell adhesion molecule (PSA-NCAM), which is specifically expressed by SVZ neuroblasts [38,39]. Thus, we queried whether TMEV infects macrophages and SVZ neuroblasts, and thereby concentrates the viral load in periventricular regions. Immunohistochemistry against the BeAn strain of virus revealed that the virus was found in and around the SVZ in regions of high CD45 activation (Fig. 7A). We immunostained for BeAn and CD45 as well as doublecortin (Dcx), a marker of SVZ neuroblasts [36,40]. Virus was associated with both CD45+ cells (not shown) and SVZ neuroblasts (Fig. 7A–C). Fig. 7D, a Z-stack of high magnification confocal microscopy optical sections shows close association between BeAn immunoreactivity and a Dcx+ neuroblast in the SVZ.

### Cuprizone induced demyelination does not induce CD45 activation in the SVZ

**Figure 8:**
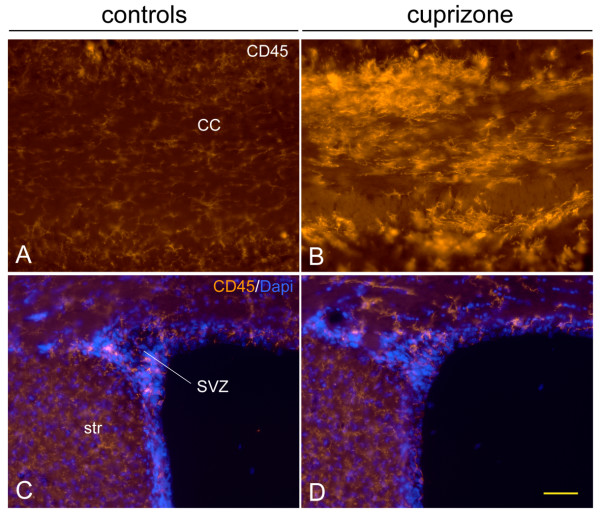
**Cuprizone demyelination does not cause CD45 activation in the SVZ.** A, B) CD45 expression is minimal in control corpus callosum (cc) but is massively activated after cuprizone. C, D) In contrast to the corpus callosum, CD45 expression remains remarkably constant in the SVZ and striatum (str) after cuprizone. Scale bar = 50 microns

We next asked whether the massive CD45 cell activation in the SVZ was general to demyelinating injuries. Cuprizone, a copper chelating agent induces demyelination in a variety of CNS regions including immediately above the SVZ, in the corpus callosum [41,42]. As expected based on known patterns of demyelination after cuprizone [43], CD45+ cells in the corpus callosum were highly activated after cuprizone (Fig. 8A,B), however, CD45 expression and cell numbers in the SVZ and periventricular striatum remained at control levels (Fig. 8C,D). This result suggests that the TMEV induced activation in the SVZ and periventricular regions is not a general response to demyelination.

### SVZ neuroblasts and emigration after TMEV infection

**Figure 9:**
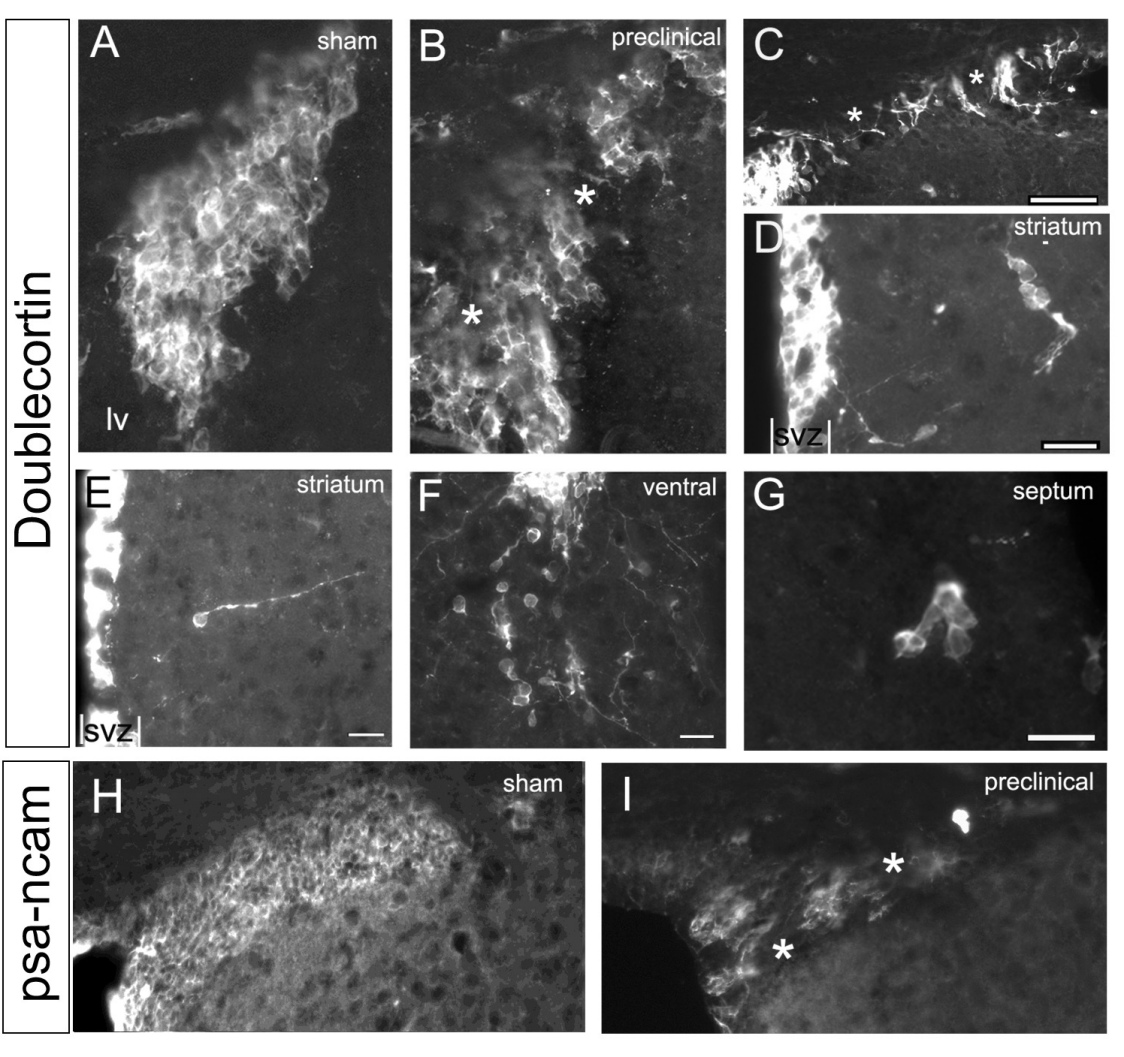
**SVZ neuroblasts after TMEV infection.** A) Dcx+ neuroblasts in control SVZ are bundled in tight clusters of chains, seen here in cross-section. B, C) Cell-cell contacts dissipate (asterisks) in the preclinical SVZ. Scale bar in C = 100 μm. D-G) examples of chains of Dcx+ cells (D, F, G) and individual Dcx+ cells emigrating from the SVZ in preclinical mice. Scale bars = 20 μm. H-I) PSA-NCAM immunohistochemistry in the dorsolateral SVZ reveals contiguous chains of cells in sham controls and loss of PSA-NCAM+ cells in preclinical mice after TMEV.

We next examined if SVZ CD45+ cell activation after TMEV infection was associated with changes in neuroblast numbers or with SVZ emigration. Striatal and hippocampal sections were labeled with anti-doublecortin (Dcx) antibodies. Dcx is a microtubule associated protein expressed by SVZ neuroblasts and is accepted as an effective read-out for SVZ neurogenesis and emigration [36,40,44,45]. In control mice, Dcx+ neuroblasts are organized in tight arrays of cells [46,47] (Fig. 9A). In infected mice, SVZ neuroblast cell-cell contacts were disrupted (Fig. 9B,C) and their membranes exhibited a loss in integrity leading to an amorphous morphology in some cells. This was especially apparent in sections that had the highest amount of CD45+ cell activation. Similar results were found with another major marker of SVZ neuroblasts; polysialylated neural cell adhesion molecule (PSA-NCAM) [38,39]. Compared to sham mice, which had contiguous arrays of PSA-NCAM+ chains, preclinical mice had significant gaps in between these chains (Fig. 9H,I). Although our results at the light microscopic level suggest loosening of neuroblast-neuroblast contacts, ultimate determination of this finding would require analysis at the electron microscope level.

Dcx immunofluorescence, Dcx+ cell numbers, and DAPI+ nuclei in the SVZ were quantified (Methods) but were not changed significantly in preclinical and early onset mice. We examined proliferation directly in the SVZ with immunohistochemistry for the G2/M cell cycle marker phosphohistone3 (PH3) [48] and found that numbers of PH3+ cells in the SVZ were not different between controls and TMEV infected preclinical and early onset mice. Though the number of Dcx+ cells, and the percent surface area occupied by Dcx immunfluorescence in the SVZ decreased at late time points, the intensity of Dcx immunofluorescence did not change appreciably at any time point. Interestingly, the number of Dcx+ cells was significantly decreased in chronic mice (38 ± 5 vs. 23 ± 5, p = 0.009), although the total number of cells (DAPI+) remained similar. This suggests that the number of Dcx+ cells decreased at late time points rather then simply the level of Dcx expression decreasing. Similar results were obtained with PSA-NCAM, with the intensity of immunofluorescence remaining similar but the surface area occupied by PSA-NCAM+ cells decreasing in the SVZ. In the hippocampal dentate gyrus (DG), there was no significant difference in Dcx+ cell counts between control and experimental animals at the first two time points. However, similar to the SVZ, there was also a statistically significant loss of DG Dcx+ cells in chronic experimental animals (24 ± 3 vs. 10 ± 2, p = 0.0007).

**Figure 10:**
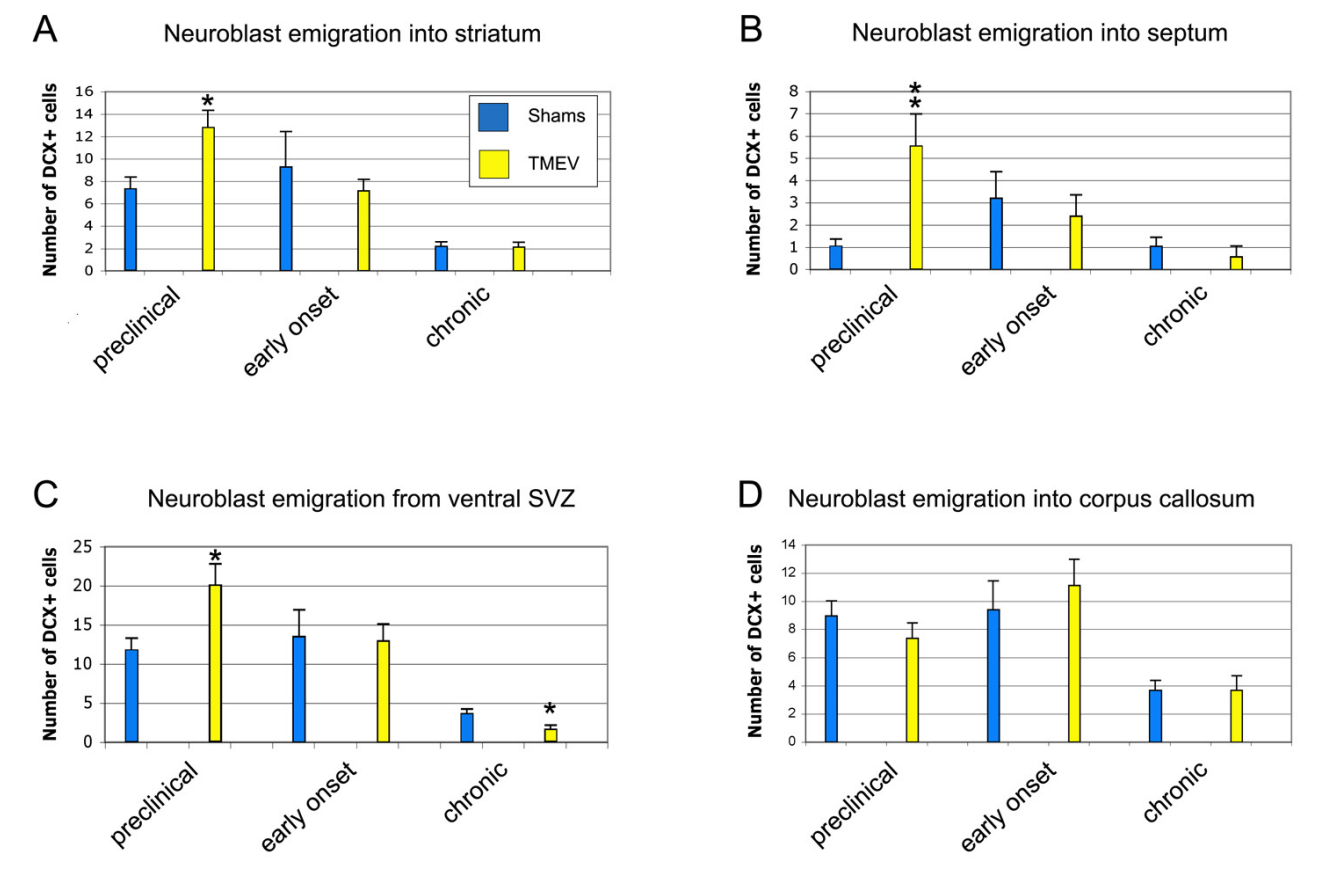
**Quantification of SVZ neuroblast numbers in the SVZ and emigration from the SVZ.** * p < 0.05, ** p < 0.01.

Small numbers of Dcx+ cells constitutively emigrate dorsally into the corpus callosum and ventrally into the nucleus accumbens and striatum [36]. Increased numbers of Dcx+ SVZ neuroblasts emigrate after a variety of insults such as stroke [13] and traumatic brain injury [49]. However it was not known if neuroblasts also emigrate into ectopic areas after TMEV infection. We found that Dcx+ cells emigrating as individuals, or in clusters of cells, were increased in mice at the time of the highest CD45+ cell activation in the SVZ (Fig. 9D–G). Preclinical experimental animals showed increased emigration into the septum, striatum, and accumbens (Fig. 9D–G; Fig. 10). In contrast, the number of Dcx+ cells in the corpus callosum were not increased (Fig. 9D; Fig. 10). By the early onset time point, numbers of emigrated cells returned to normal (Fig. 9A–C; Fig. 10). Unexpectedly, the numbers of ventrally emigrating cells was slightly decreased in chronic mice (Fig. 9C; Fig. 10), matching the loss of Dcx+ cell numbers in the SVZ at that time point. These results suggests that TMEV causes SVZ neuroblasts to emigrate into grey matter at preclinical stages of the disease.

## Discussion

We showed that in TMEV-induced inflammation, CD45+ cell activation within the forebrain is most consistently found in the SVZ and periventricular regions. Interestingly, neuroblast emigration from the SVZ increased at the same time as CD45+ cell activation. At later time points, when SVZ inflammation had subsided, neurogenesis decreased. We also showed that the forebrain is affected before the cerebellum and spinal cord. In contrast to the spinal cord, where extensive CD45+ cell infiltration was observed in white matter tracts, forebrain white matter was relatively spared at all time points.

A wide variety of preclinical models of injury and disease increase neurogenesis and induce neuroblast emigration from the SVZ [1,12,13,49]. We showed that TMEV induced SVZ neuroblasts to emigrate, to our knowledge, the first example of neuronal emigration from the SVZ in a model of brain inflammation. Since maximal emigration occurred at the same time as maximal CD45+ cell activation in the forebrain, it is very likely that CD45+ activated cells secrete molecules contributing to SVZ neuroblast emigration. Future mechanistic studies may identify molecular interactions between CD45+ cells and SVZ neuroblasts and thereby lead to molecular approaches designed to augment neural repair.

Although neuroblast emigration was augmented at early time points after TMEV inoculation, the number of neuroblasts was unchanged. Other studies have shown that inflammation rapidly reduces adult neurogenesis [5,6], however we did not observe decreases in Dcx+ neuroblasts during the time of maximal inflammation. Decreased numbers of Dcx+ neuroblasts were only found in "chronic mice", well after major inflammation had subsided. Surprisingly, Dcx+ cell numbers decreased in both the SVZ and dentate gyrus, even though only the former exhibited focal inflammation. Our results suggest that even though SVZ neuroblasts emigrate relatively soon after TMEV inoculation, any autologous repair may be attenuated by delayed decreases in neurogenesis.

One of the most novel and unpredictable findings of our study was that although forebrain activation of CD45+ cells was stochastic from section to section, and from mouse to mouse, it was remarkably consistent in the SVZ. Our results suggest that this is not a general response to demyelination since cuprizone induced demyelination did not activate CD45 expression in the SVZ. We favor two main possible mechanisms for our finding after TMEV infection: viral SVZ macrophage tropism or viral spread through the ventricular system. CD45+ cells are constitutively semi-activated in the SVZ [7] and CD45+ cells were consistently activated in the SVZ and in the RMS. Since TMEV preferentially infects activated microglia [50,51], the relatively high levels of constitutive macrophage activation in the SVZ [7] may predispose them to infection. Another interpretation of our study is that the ventricular system is a conduit for viral spread. CD45 activation was high in the preclinical group near the ventricular system: lateral ventricle, 3^rd ^ventricle, periventricular hippocampus, and 4^th ^ventricle. Immunodetectable virus was associated with SVZ neuroblasts and Theiler's virus exhibits tropism for sialic acid residues [26], thus it also possible that PSA-NCAM+ neuroblasts may be preferentially infected.

We found vigorous CD45+ cell activation throughout the forebrain already at two weeks after viral induction, suggesting that it commenced earlier. Microglial activation can occur quite rapidly, within hours to days after insults. By the time clinical symptomatology appeared in TMEV mice, CD45+ cell activation in the forebrain had diminished. In contrast to the forebrain, the major CD45+ cell activation in the cerebellum occurred three months after TMEV infection, which is surprising since TMEV was injected into the cerebellum. Similar to the cerebellum, the major spinal cord response was in the chronic group. Based on our results with this model, subtle forebrain inflammation mediated symptomatology may precede the severe spinal cord mediated motor defects in MS.

Whereas the large majority of newborn cells in the adult SVZ are young neurons, a few oligodendrocytes are also generated constitutively [12,52]. EAE induces increased numbers of oligodendrocyte genesis and emigration out of the SVZ [28,29,53]. Interestingly postmortem human studies suggest that SVZ cells, probably glioblasts, emigrate into adjacent regions in MS [54]. The effects of preclinical models of MS on neurogenesis and neuroblast emigration were less clear, especially with regards to TMEV. This was important to address since, in addition to glial loss, a significant amount of neuronal loss occurs in MS [19]. Our results show that even though neurons are lost in MS, neurogenic regions of the brain do not increase the production of newborn neurons. This is unlike multiple other models of neuronal loss such as stroke which generally increase neurogenesis [1,13].

An interesting clinical observation is that MS is frequently associated with periventricular lesions; "Dawson's fingers" [31]. As seen with MRI, these lesions extend from the ependyma into the corpus callosum and other brain regions [32]. The lesions course along subventricular zone (subependymal) blood vessels, suggesting they are routes of entry for destructive immune cells. As in Dawson's fingers, CD45+ cell activation was frequently associated with SVZ blood vessels and it may be that these were infiltrating macrophages. Recent studies have documented increased cell density and proliferation in the SVZ of MS patients [54]. These observations are compatible with the massive CD45+ cell activation we consistently observed around the lateral ventricles. High numbers of CD45+ inflammatory cells concentrate in the circumventricular organs, during EAE [55] and MS cerebral hemisphere lesions are primarily perivascular [56,57]. Regardless of the mechanism, an increasing body of evidence suggests that periventricular regions, including the neurogenic SVZ, are particularly affected in preclinical models and human MS.

## List of abbreviations

CC: corpus callosum; C: chronic; DAPI: 4',6-diamidino-2-phenylindole; EAE: experimental allergic encephalomyelitis; EO: early onset; LV: lateral ventricle; MS: multiple sclerosis; PBS: phosphate buffered saline; PC: preclinical; RMS: rostral migratory stream; SGZ: subgranuar zone; SVZ: subventricular zone; TMEV: Theiler's murine encephalomyelitis virus.

## Competing interests

The authors declare that they have no competing interests.

## Authors' contributions

GEG. Sectioned brains, performed the majority of immunohistochemistry, analyzed sections, and wrote the manuscript. AG sectioned brains, performed immunohistochemistry, and analyzed sections. REJ performed immunohistochemsitry and analyzed sections. LKFA examined the cuprizone model. WSB performed TMEV inoculations. SDM provided expertise on the TMEV model and advised on experimental design and the manuscript. FGS provided expertise on the subventricular zone and wrote the manuscript.

## References

[B1] Dizon MLV, Szele FG, Levison SW (2005). The subventricular zone responds dynamically to mechanical brain injuries. Mammalian Subventricular Zones: Their Roles In Brain Development, Cell Replacement, And Disease.

[B2] Gage FH (2000). Mammalian neural stem cells. Science.

[B3] Altman J (1969). Autoradiographic and histological studies of postnatal neurogenesis. IV. Cell proliferation and migration in the anterior forebrain, with special reference to persisting neurogenesis in the olfactory bulb. J Comp Neurol.

[B4] Reynolds BA, Weiss S (1992). Generation of neurons and astrocytes from isolated cells of the adult mammalian central nervous system. Science.

[B5] Ekdahl CT, Claasen J-H, Bonde S, Kokaia Z, Lindvall O (2003). Inflammation is detrimental for neurogenesis in adult brain. PNAS.

[B6] Monje ML, Toda H, Palmer TD (2003). Inflammatory Blockade Restores Adult Hippocampal Neurogenesis. Science.

[B7] Goings GE, Kozlowski DA, Szele FG (2006). Differential activation of microglia in neurogenic versus non-neurogenic regions of the forebrain. Glia.

[B8] Cammermeyer J (1965). The hypependymal microglia cell. Z Anat Entwicklungsgesch.

[B9] Lewis PD (1968). The fate of the subependymal cell in the adult rat brain, with a note on the origin of microglia. Brain.

[B10] Mohri I, Eguchi N, Suzuki K, Urade Y, Taniike M (2003). Hematopoietic prostaglandin D synthase is expressed in microglia in the developing postnatal mouse brain. Glia.

[B11] Levison SW, Druckman SK, Young GM, Basu A (2003). Neural stem cells in the subventricular zone are a source of astrocytes and oligodendrocytes, but not microglia. Dev Neurosci.

[B12] Goings GE, Sahni V, Szele FG (2004). Migration patterns of subventricular zone cells in adult mice change after cerebral cortex injury. Brain Res.

[B13] Arvidsson A, Collin T, Kirik D, Kokaia Z, Lindvall O (2002). Neuronal replacement from endogenous precursors in the adult brain after stroke. Nat Med.

[B14] Townsend KP, Vendrame M, Ehrhart J, Faza B, Zeng J, Town T, Tan J (2004). CD45 isoform RB as a molecular target to oppose lipopolysaccharide-induced microglial activation in mice. Neurosci Lett.

[B15] Tan J, Town T, Mori T, Wu Y, Saxe M, Crawford F, Mullan M (2000). CD45 opposes beta-amyloid peptide-induced microglial activation via inhibition of p44/42 mitogen-activated protein kinase. J Neurosci.

[B16] Tan J, Town T, Mullan M (2000). CD45 inhibits CD40L-induced microglial activation via negative regulation of the Src/p44/42 MAPK pathway. J Biol Chem.

[B17] Sedgwick JD, Schwender S, Imrich H, Dorries R, Butcher GW, ter Meulen V (1991). Isolation and direct characterization of resident microglial cells from the normal and inflamed central nervous system. Proc Natl Acad Sci USA.

[B18] Penninger JM, Irie-Sasaki J, Sasaki T, Oliveira-dos-Santos AJ (2001). CD45: new jobs for an old acquaintance. Nat Immunol.

[B19] Diem R, Sattler MB, Bahr M (2006). Neurodegeneration and -protection in autoimmune CNS inflammation. J Neuroimmunol.

[B20] Meyer R, Weissert R, Diem R, Storch MK, de Graaf KL, Kramer B, Bahr M (2001). Acute neuronal apoptosis in a rat model of multiple sclerosis. J Neurosci.

[B21] Wegner C, Esiri MM, Chance SA, Palace J, Matthews PM (2006). Neocortical neuronal, synaptic, and glial loss in multiple sclerosis. Neurology.

[B22] dal Canto MC, Lipton HL (1977). A new model of persistent viral infection with primary demyelination. Neurol Neurocir Psiquiatr.

[B23] Miller SD, Vanderlugt CL, Begolka WS, Pao W, Yauch RL, Neville KL, Katz-Levy Y, Carrizosa A, Kim BS (1997). Persistent infection with Theiler's virus leads to CNS autoimmunity via epitope spreading. Nat Med.

[B24] Mack CL, Vanderlugt-Castaneda CL, Neville KL, Miller SD (2003). Microglia are activated to become competent antigen presenting and effector cells in the inflammatory environment of the Theiler's virus model of multiple sclerosis. J Neuroimmunol.

[B25] Fuller KG, Olson JK, Howard LM, Croxford JL, Miller SD (2004). Mouse models of multiple sclerosis: experimental autoimmune encephalomyelitis and Theiler's virus-induced demyelinating disease. Methods Mol Med.

[B26] Lipton HL, Kumar AS, Hertzler S, Reddi HV (2006). Differential usage of carbohydrate co-receptors influences cellular tropism of Theiler's murine encephalomyelitis virus infection of the central nervous system. Glycoconj J.

[B27] Ono K, Tomasiewicz H, Magnuson T, Rutishauser U (1994). N-CAM mutation inhibits tangential neuronal migration and is phenocopied by enzymatic removal of polysialic acid. Neuron.

[B28] Nait-Oumesmar B, Decker L, Lachapelle F, Avellana-Adalid V, Bachelin C, Van Evercooren AB (1999). Progenitor cells of the adult mouse subventricular zone proliferate, migrate and differentiate into oligodendrocytes after demyelination. Eur J Neurosci.

[B29] Picard-Riera N, Decker L, Delarasse C, Goude K, Nait-Oumesmar B, Liblau R, Pham-Dinh D, Evercooren AB (2002). Experimental autoimmune encephalomyelitis mobilizes neural progenitors from the subventricular zone to undergo oligodendrogenesis in adult mice. Proc Natl Acad Sci USA.

[B30] Pluchino S, Quattrini A, Brambilla E, Gritti A, Salani G, Dina G, Galli R, Del Carro U, Amadio S, Bergami A, Furlan R, Comi G, Vescovi AL, Martino G (2003). Injection of adult neurospheres induces recovery in a chronic model of multiple sclerosis. Nature.

[B31] Dawson JD (1916). The Histology of Disseminated Multiple Sclerosis. Transactions of the Royal Society of Edinburgh.

[B32] Olek MJ (2005). Multiple sclerosis : etiology, diagnosis, and new treatment strategies.

[B33] Paxinos G, Franklin KBJ (2001). The mouse brain in stereotaxic coordinates.

[B34] Bailey SL, Schreiner B, McMahon EJ, Miller SD (2007). CNS myeloid DCs presenting endogenous myelin peptides 'preferentially' polarize CD4(+) T(H)-17 cells in relapsing EAE. Nat Immunol.

[B35] Bulloch K, Miller MM, Gal-Toth J, Milner TA, Gottfried-Blackmore A, Waters EM, Kaunzner UW, Liu K, Lindquist R, Nussenzweig MC, Steinman RM, McEwen BS (2008). CD11c/EYFP transgene illuminates a discrete network of dendritic cells within the embryonic, neonatal, adult, and injured mouse brain. J Comp Neurol.

[B36] Yang HKC, Sundholm-Peters NL, Goings G, Walker AS, Hyland K, Szele F (2004). Distribution of doublecortin expressing cells near the lateral ventricles in the adult mouse brain. J Neurosci Res.

[B37] Lipton HL, Twaddle G, Jelachich ML (1995). The predominant virus antigen burden is present in macrophages in Theiler's murine encephalomyelitis virus-induced demyelinating disease. J Virol.

[B38] Szele FG, Dowling JJ, Gonzales C, Theveniau M, Rougon G, Chesselet MF (1994). Pattern of expression of highly polysialylated neural cell adhesion molecule in the developing and adult rat striatum. Neuroscience.

[B39] Rousselot P, Lois C, Alvarez-Buylla A (1995). Embryonic (PSA) N-CAM reveals chains of migrating neuroblasts between the lateral ventricle and the olfactory bulb of adult mice. J Comp Neurol.

[B40] Brown JP, Couillard-Despres S, Cooper-Kuhn CM, Winkler J, Aigner L, Kuhn HG (2003). Transient expression of doublecortin during adult neurogenesis. J Comp Neurol.

[B41] McMahon EJ, Suzuki K, Matsushima GK (2002). Peripheral macrophage recruitment in cuprizone-induced CNS demyelination despite an intact blood-brain barrier. J Neuroimmunol.

[B42] Torkildsen O, Brunborg LA, Myhr KM, Bo L (2008). The cuprizone model for demyelination. Acta Neurol Scand Suppl.

[B43] Sun SW, Liang HF, Trinkaus K, Cross AH, Armstrong RC, Song SK (2006). Noninvasive detection of cuprizone induced axonal damage and demyelination in the mouse corpus callosum. Magn Reson Med.

[B44] Gleeson JG, Lin PT, Flanagan LA, Walsh CA (1999). Doublecortin is a microtubule-associated protein and is expressed widely by migrating neurons. Neuron.

[B45] Couillard-Despres S, Winner B, Schaubeck S, Aigner R, Vroemen M, Weidner N, Bogdahn U, Winkler J, Kuhn HG, Aigner L (2005). Doublecortin expression levels in adult brain reflect neurogenesis. Eur J Neurosci.

[B46] Thomas LB, Gates MA, Steindler DA (1996). Young neurons from the adult subependymal zone proliferate and migrate along an astrocyte, extracellular matrix-rich pathway. Glia.

[B47] Doetsch F, Alvarez-Buylla A (1996). Network of tangential pathways for neuronal migration in adult mammalian brain. Proc Natl Acad Sci USA.

[B48] Eisch AJ, Mandyam CD (2007). Adult neurogenesis: can analysis of cell cycle proteins move us "Beyond BrdU"?. Curr Pharm Biotechnol.

[B49] Sundholm-Peters NL, Yang HK, Goings GE, Walker AS, Szele FG (2005). Subventricular zone neuroblasts emigrate toward cortical lesions. J Neuropathol Exp Neurol.

[B50] Jelachich ML, Bandyopadhyay P, Blum K, Lipton HL (1995). Theiler's virus growth in murine macrophage cell lines depends on the state of differentiation. Virology.

[B51] Jelachich ML, Bramlage C, Lipton HL (1999). Differentiation of M1 myeloid precursor cells into macrophages results in binding and infection by Theiler's murine encephalomyelitis virus and apoptosis. J Virol.

[B52] Menn B, Garcia-Verdugo JM, Yaschine C, Gonzalez-Perez O, Rowitch D, Alvarez-Buylla A (2006). Origin of oligodendrocytes in the subventricular zone of the adult brain. J Neurosci.

[B53] Calza L, Giardino L, Pozza M, Bettelli C, Micera A, Aloe L (1998). Proliferation and phenotype regulation in the subventricular zone during experimental allergic encephalomyelitis: in vivo evidence of a role for nerve growth factor. Proc Natl Acad Sci USA.

[B54] Nait-Oumesmar B, Picard-Riera N, Kerninon C, Decker L, Seilhean D, Hoglinger GU, Hirsch EC, Reynolds R, Baron-Van Evercooren A (2007). Activation of the subventricular zone in multiple sclerosis: evidence for early glial progenitors. Proc Natl Acad Sci USA.

[B55] Schulz M, Engelhardt B (2005). The circumventricular organs participate in the immunopathogenesis of experimental autoimmune encephalomyelitis. Cerebrospinal Fluid Res.

[B56] Oleszak EL, Chang JR, Friedman H, Katsetos CD, Platsoucas CD (2004). Theiler's virus infection: a model for multiple sclerosis. Clin Microbiol Rev.

[B57] Barkhof F, Filippi M, Miller DH, Scheltens P, Campi A, Polman CH, Comi G, Ader HJ, Losseff N, Valk J (1997). Comparison of MRI criteria at first presentation to predict conversion to clinically definite multiple sclerosis. Brain.

